# Supplementary material for: Structural basis of the P4B ATPase lipid flippase activity
Source: Nat Commun. 2021 Oct 13;12:5963. doi: 10.1038/s41467-021-26273-0 (PMC8514546; doi:10.1038/s41467-021-26273-0)
Supplement: Supplementary file 1 — Supplementary Information [file 41467_2021_26273_MOESM1_ESM.pdf]

Supplemental information for

Structural basis of the P4B ATPase lipid flippase activity

Lin Bai, Bhawik K. Jain, Qinglong You, H. Diessel Duan, Mehmet Takar, Todd R. Graham, and Huilin Li

This document contains:

Three supplemental tables

Ten supplemental figures

Supplemental Table 1. Cryo-EM data collection, refinement, and validations

	Neo1		
	**E1-ATP**	**E2P-transition**	**E2P**
**Data collection and processing**			
Magnification (k)	130	130	130
Voltage (kV)	300	300	300
Electron exposure ($e^-/Å^2$)	60	60	60
Defocus range (-μm)	1.5-3.0	1.5-3.0	1.5-3.0
Pixel size (Å)	0.826	0.826	0.826
Symmetry imposed	C1	C1	C1
Micrograph #	1175	5119	4354
Initial particle #	668,445	3,037,926	2,315,630
Final particle #	264,891	1,673,321	1,279,510
Map resolution (Å)	5.64	3.08	3.25
FSC threshold	0.143	0.143	0.143
**Refinement**			
Map sharpening *B* factor ($Å^2$)	-168	-153	-275
Model composition			
Non-hydrogen atoms	7426	7400	7399
Protein residues	929	929	929
B factors ($Å^2$)			
Protein	285.56	58.19	65.26
Ligand	327.71	44.13	52.07
R.m.s. deviations			
Bond lengths (Å)	0.003	0.004	0.004
Bond angles (°)	0.863	0.815	0.769
Validation			
MolProbity score	1.99	1.93	2.23
Clashscore	11.10	8.78	8.85
Poor rotamers (%)	0	0	0
Ramachandran plot			
Favored (%)	93.47	92.82	93.47
Allowed (%)	6.53	7.18	6.53
Disallowed (%)	0	0	0

Supplemental Table 2. Primers used in the study

Primer name	Sequence
Neo1 Q193A FP	ACCCTATTATATGAAGCCTTCAAATTCTTTTAC
Neo1 Q193A RP	GTAAAAGAATTTGAAGGCTTCATATAATAGGGT
Neo1 S221L FP	ATAGGGTATTTATCATTATATATTGTACCTTTA
Neo1 S221L RP	TAAAGGTACAATATATAATGATAAATACCCTAT
Neo1 S221A FP	ATAGGGTATTTATCAGCCTATATTGTACCTTTA
Neo1 S221A RP	TAAAGGTACAATATAGGCTGATAAATACCCTAT
Neo1 K236A FP	ACAGTAACCATGGCAGCCGAAGCTATAGATGAT
Neo1 K236A RP	ATCATCTATAGCTTCGGCTGCCATGGTTACTGT
Neo1 K236R FP	ACAGTAACCATGGCAAGAGAAGCTATAGATGAT
Neo1 K236R RP	ATCATCTATAGCTTCTCTTGCCATGGTTACTGT
Neo1 E237A FP	GTAACCATGGCAAAGGCCGCTATAGATGATATT
Neo1 E237A RP	AATATCATCTATAGCGGCCTTTGCCATGGTTAC
Neo1 P456A FP	TTTTCTACCATCATCGCCGTCTCTTTAAGAGTT
Neo1 P456A RP	AACTCTTAAAGAGACGGCGATGATGGTAGAAAA
Neo1 P456G FP	TTTTCTACCATCATCGGGGTCTCTTTAAGAGTT
Neo1 P456G RP	AACTCTTAAAGAGACCCCGATGATGGTAGAAAA
Neo1 T453A FP	CTGATATTGTTTTCTGCCATCATCCCTGTCTCT
Neo1 T453A RP	AGAGACAGGGATGATGGCAGAAAACAATATCAG
Neo1 T453S FP	CTGATATTGTTTTCTTCTATCATCCCTGTCTCT
Neo1 T453S RP	AGAGACAGGGATGATAGAAGAAAACAATATCAG
Neo1 Q243AR244A FP	GAAGCTATAGATGATATTGCCGCCAGGAGACGTGATAGA
Neo1 Q243AR244A RP	TCTATCACGTCTCCTGGCGGCAATATCATCTATAGCTTC
Neo1 R247A FP	ATTCAGAGAAGGAGAGCCGATAGAGAGTCAAAC
Neo1 R247A RP	GTTTGACTCTCTATCGGCTCTCCTTCTCTGAAT
Neo1 R247L FP	ATTCAGAGAAGGAGATTAGATAGAGAGTCAAAC
Neo1 R247L RP	GTTTGACTCTCTATCTAATCTCCTTCTCTGAAT
Neo1 R460A FP	ATCCCTGTCTCTTTAGCCGTTAATTTAGATTTA
Neo1 R460A RP	TAAATCTAAATTAACGGCTAAAGAGACAGGGAT
Neo1 R460Y FP	ATCCCTGTCTCTTTATATGTTAATTTAGATTTA
Neo1 R460Y RP	TAAATCTAAATTAACATATAAAGAGACAGGGAT
Neo1 S488A FP	ACAATTGTTAGAACGGCCACAATTCCAGAGGAT
Neo1 S488A RP	ATCCTCTGGAATTGTGGCCGTTCTAACAATTGT
Neo1 S488W FP	ACAATTGTTAGAACGTGGACAATTCCAGAGGAT
Neo1 S488W RP	ATCCTCTGGAATTGTCCACGTTCTAACAATTGT
Neo1 Q209G FP	GTAGTTGCATTATCGGGAGCCGTTCCTGCACTA
Neo1 Q209G RP	TAGTGCAGGAACGGCTCCCGATAATGCAACTAC
Neo1 S452Q FP	TATCTGATATTGTTTCAGACCATCATCCCTGTC
Neo1 S452Q RP	GACAGGGATGATGGTCTGAAACAATATCAGATA
Neo1 E237D FP	GTAACCATGGCAAAGGATGCTATAGATGATATT
Neo1 E237D RP	AATATCATCTATAGCATCCTTTGCCATGGTTAC

Supplemental Table 3. Yeast strains and plasmids used in the study

Strain	Genotype	Plasmid	Source
MTY10-TAPFL-001	*(YWY10)neo1Δ+pRS423-GPD-TAP-3XFLAG-NEO1*	*pRS423-GPD-TAP-3XFLAG-NEO1*	This study
MTY20-TAPFL-001	*(YWY20)neo1Δ+pRS423-GPD-TAP-3XFLAG-NEO1*	*pRS423-GPD-TAP-3XFLAG-NEO1*	This study
MTY10/20-dp-TAPFL-001	*(YWY10*YWY20)neo1Δ/neo1Δ+pRS423-GPD-TAP-3XFLAG-NEO1*	*pRS423-GPD-TAP-3XFLAG-NEO1*	This study
YWY10	MAT**a** *his3Δ1 leu2Δ0 ura3Δ0 lys2Δ0 neo1Δ::KanMX*	pRS416-NEO1	[38]
BKJ401	YWY10	pRS313-NEO1	This study
BKJ402	YWY10	pRS313	This study
BKJ403	YWY10	pRS313-NEO1 Q209G	This study
BKJ404	YWY10	pRS313-Neo1 S221A	This study
BKJ405	YWY10	pRS313-Neo1 S221L	This study
BKJ406	YWY10	pRS313-Neo1 S452A	This study
BKJ407	YWY10	pRS313-Neo1 S452Q	This study
BKJ408	YWY10	pRS313-Neo1 T453A	This study
BKJ409	YWY10	pRS313-Neo1 T453S	This study
BKJ410	YWY10	pRS313-Neo1 K236A	This study
BKJ411	YWY10	pRS313-Neo1 K236R	This study
BKJ412	YWY10	pRS313-Neo1 E237A	This study
BKJ413	YWY10	pRS313-Neo1 E237D	This study
BKJ414	YWY10	pRS313-Neo1 R247A	This study
BKJ415	YWY10	pRS313-Neo1 R247L	This study
BKJ416	YWY10	pRS313-Neo1 S488A	This study
BKJ417	YWY10	pRS313-Neo1 S488W	This study
BKJ418	YWY10	pRS313-Neo1 Q193A	This study
BKJ419	YWY10	pRS313-Neo1 QR-AA	This study
BKJ420	YWY10	pRS313-Neo1 P456A	This study
BKJ421	YWY10	pRS313-Neo1 P456G	This study
BKJ422	YWY10	pRS313-Neo1 R460A	This study
BKJ423	YWY10	pRS313-Neo1 R460Y	This study
BKJ424	MAT**a** *his3Δ1 leu2Δ0 ura3Δ0 lys2Δ0 neo1Δ::KanMX*	pRS313-NEO1	This study
BKJ425	MAT**a** *his3Δ1 leu2Δ0 ura3Δ0 lys2Δ0 neo1Δ::KanMX*	pRS313	This study
BKJ426	MAT**a** *his3Δ1 leu2Δ0 ura3Δ0 lys2Δ0 neo1Δ::KanMX*	pRS313-NEO1 Q209G	This study
BKJ427	MAT**a** *his3Δ1 leu2Δ0 ura3Δ0 lys2Δ0 neo1Δ::KanMX*	pRS313-Neo1 S221A	This study
BKJ428	MAT**a** *his3Δ1 leu2Δ0 ura3Δ0 lys2Δ0 neo1Δ::KanMX*	pRS313-Neo1 S221L	This study
BKJ429	MAT**a** *his3Δ1 leu2Δ0 ura3Δ0 lys2Δ0 neo1Δ::KanMX*	pRS313-Neo1 S452A	This study

BKJ430	MAT**a** *his3Δ1 leu2Δ0 ura3Δ0 lys2Δ0 neo1Δ::KanMX*	pRS313-Neo1 S452Q	This study
BKJ431	MAT**a** *his3Δ1 leu2Δ0 ura3Δ0 lys2Δ0 neo1Δ::KanMX*	pRS313-Neo1 T453A	This study
BKJ432	MAT**a** *his3Δ1 leu2Δ0 ura3Δ0 lys2Δ0 neo1Δ::KanMX*	pRS313-Neo1 T453S	This study
BKJ433	MAT**a** *his3Δ1 leu2Δ0 ura3Δ0 lys2Δ0 neo1Δ::KanMX*	pRS313-Neo1 K236A	This study
BKJ434	MAT**a** *his3Δ1 leu2Δ0 ura3Δ0 lys2Δ0 neo1Δ::KanMX*	pRS313-Neo1 K236R	This study
BKJ435	MAT**a** *his3Δ1 leu2Δ0 ura3Δ0 lys2Δ0 neo1Δ::KanMX*	pRS313-Neo1 E237A	This study
BKJ436	MAT**a** *his3Δ1 leu2Δ0 ura3Δ0 lys2Δ0 neo1Δ::KanMX*	pRS313-Neo1 E237D	This study
BKJ437	MAT**a** *his3Δ1 leu2Δ0 ura3Δ0 lys2Δ0 neo1Δ::KanMX*	pRS313-Neo1 R247A	This study
BKJ438	MAT**a** *his3Δ1 leu2Δ0 ura3Δ0 lys2Δ0 neo1Δ::KanMX*	pRS313-Neo1 R247L	This study
BKJ439	MAT**a** *his3Δ1 leu2Δ0 ura3Δ0 lys2Δ0 neo1Δ::KanMX*	pRS313-Neo1 S488A	This study
BKJ440	MAT**a** *his3Δ1 leu2Δ0 ura3Δ0 lys2Δ0 neo1Δ::KanMX*	pRS313-Neo1 S488W	This study
BKJ441	MAT**a** *his3Δ1 leu2Δ0 ura3Δ0 lys2Δ0 neo1Δ::KanMX*	pRS313-Neo1 Q193A	This study
BKJ442	MAT**a** *his3Δ1 leu2Δ0 ura3Δ0 lys2Δ0 neo1Δ::KanMX*	pRS313-Neo1 QR-AA	This study
BKJ443	MAT**a** *his3Δ1 leu2Δ0 ura3Δ0 lys2Δ0 neo1Δ::KanMX*	pRS313-Neo1 P456A	This study
BKJ444	MAT**a** *his3Δ1 leu2Δ0 ura3Δ0 lys2Δ0 neo1Δ::KanMX*	pRS313-Neo1 R460A	This study
BKJ445	MAT**a** *his3Δ1 leu2Δ0 ura3Δ0 lys2Δ0 neo1Δ::KanMX*	pRS313-Neo1 R460Y	This study
MTY10-615M2D	YWY10 *drs2Δ::HygR*	pRS416-NEO1	[68]
BKJ446	MTY10-615M2D	pRS313-NEO1	This study
BKJ447	MTY10-615M2D	pRS313	This study
BKJ448	MTY10-615M2D	pRS313-NEO1 Q209G	This study
BKJ449	MTY10-615M2D	pRS313-Neo1 S221A	This study
BKJ450	MTY10-615M2D	pRS313-Neo1 S221L	This study
BKJ451	MTY10-615M2D	pRS313-Neo1 S452A	This study
BKJ452	MTY10-615M2D	pRS313-Neo1 S452Q	This study
BKJ453	MTY10-615M2D	pRS313-Neo1 T453A	This study
BKJ454	MTY10-615M2D	pRS313-Neo1 T453S	This study
BKJ455	MTY10-615M2D	pRS313-Neo1 K236A	This study
BKJ456	MTY10-615M2D	pRS313-Neo1 K236R	This study
BKJ457	MTY10-615M2D	pRS313-Neo1 E237A	This study
BKJ458	MTY10-615M2D	pRS313-Neo1 E237D	This study
BKJ459	MTY10-615M2D	pRS313-Neo1 R247A	This study
BKJ460	MTY10-615M2D	pRS313-Neo1 R247L	This study

BKJ461	MTY10-615M2D	pRS313-Neo1 S488A	This study
BKJ462	MTY10-615M2D	pRS313-Neo1 S488W	This study
BKJ463	MTY10-615M2D	pRS313-Neo1 Q193A	This study
BKJ464	MTY10-615M2D	pRS313-Neo1 QR-AA	This study
BKJ465	MTY10-615M2D	pRS313-Neo1 P456A	This study
BKJ466	MTY10-615M2D	pRS313-Neo1 P456G	This study
BKJ467	MTY10-615M2D	pRS313-Neo1 R460A	This study
BKJ468	MTY10-615M2D	pRS313-Neo1 R460Y	This study
JH59	MAT**α** *his3 leu2 ura3 lys2 AUR1-mKate::URA3*		[57]
BKJ469	JH59	pRS413-pADH-GFP-Neo1	This study
BKJ470	JH59	pRS413-pADH-GFP-Neo1 Q193A	This study
BKJ471	JH59	pRS413-pADH-GFP-Neo1 Q209G	This study
BKJ472	JH59	pRS413-pADH-GFP-Neo1 S452Q	This study
BKJ473	JH59	pRS413-pADH-GFP-Neo1 R247L	This study
BKJ474	JH59	pRS413-pADH-GFP-Neo1 E237D	This study
BKJ475	JH59	pRS413-pADH-GFP-Neo1 P456A	This study
BKJ476	JH59	pRS413-pADH-GFP-Neo1 S488A	This study
BKJ477	JH59	pRS413-pADH-GFP-Neo1 R247L	This study
BY4741	MAT**a** *his3Δ1 leu2Δ0 met15Δ0 ura3Δ0*		Invitrogen

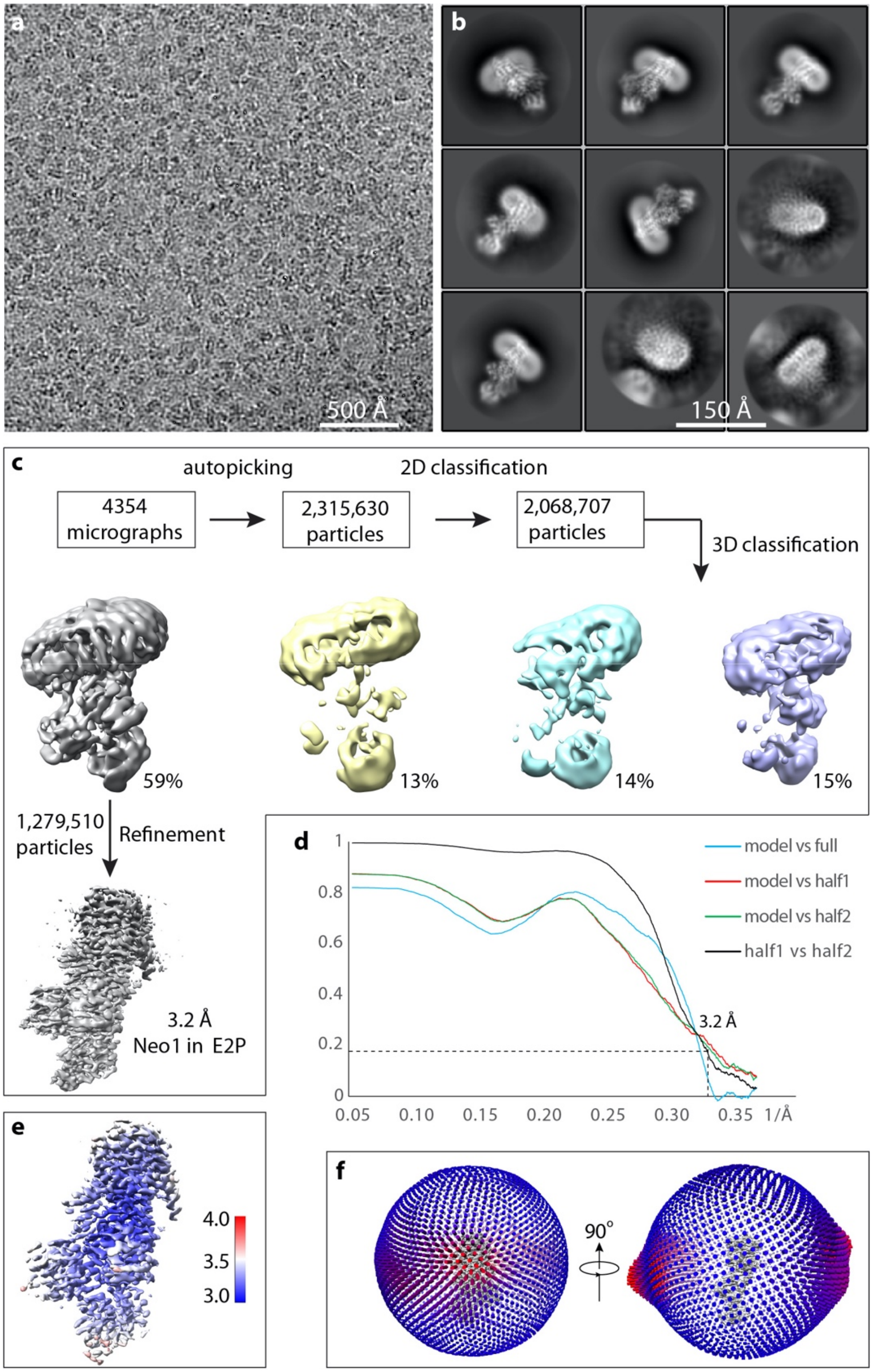

Supplemental Figure 1. Cryo-EM data processing and resolution estimation of Neo1 in the E2P state. a) A representative electron micrograph. **b**) Selected reference-free 2D class averages. **c**) Cryo-EM data processing procedure. **d**) Gold-standard Fourier shell correlations. **e**) Color-coded local resolution map of the final 3D map. **f**) Angular distribution of raw particles used in the final reconstruction of the 3.2-Å 3D map.

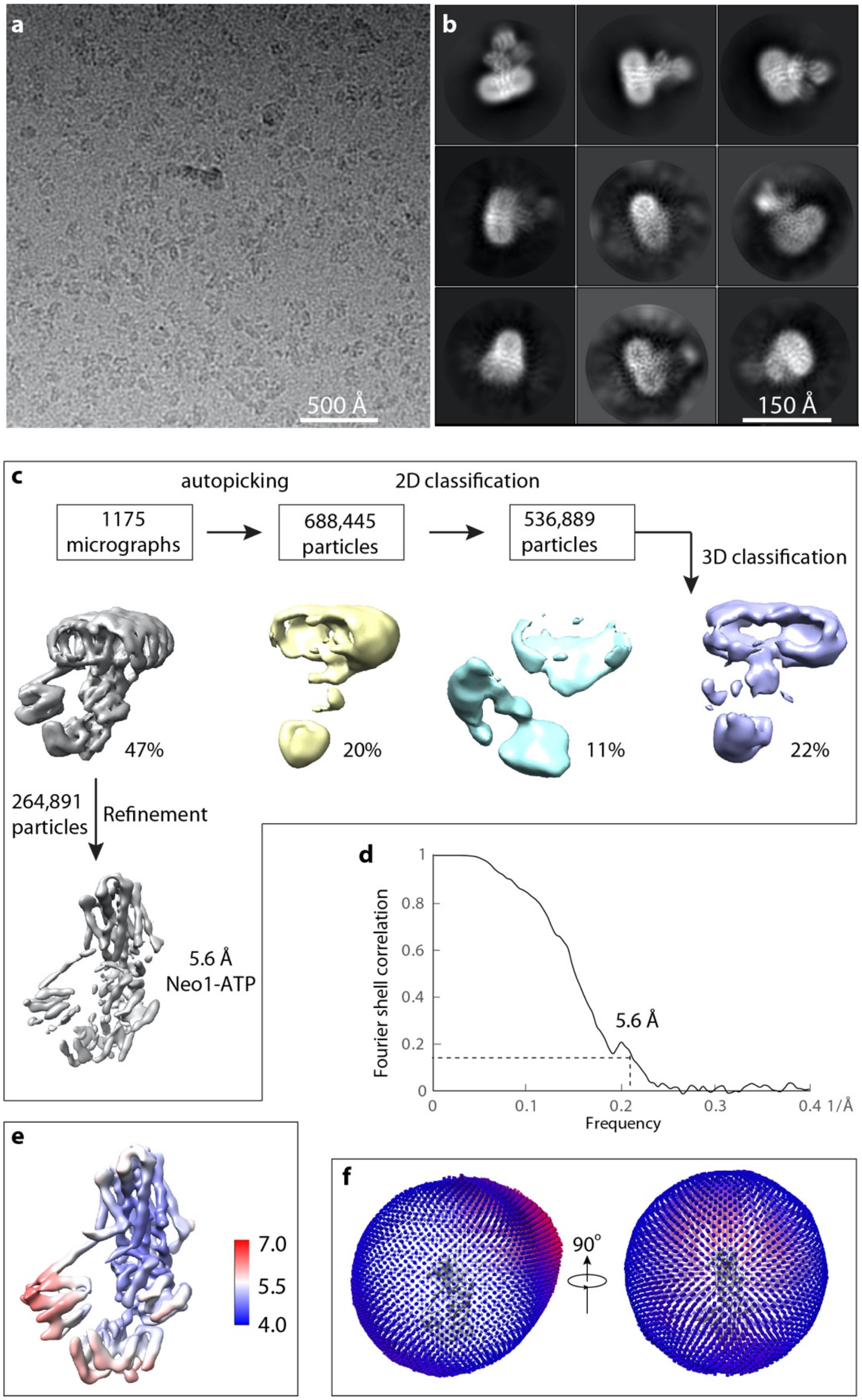

Supplemental Figure 2. Cryo-EM data processing and resolution estimation of Neo1 in the E1-ATP state. a) A representative electron micrograph. **b**) Selected reference-free 2D class averages. **c**) Cryo-EM data processing procedure. **d**) Gold-standard Fourier shell correlations. **e**) Color-coded local resolution map of the final 3D map. **f**) Angular distribution of raw particles used in the final reconstruction of the 5.6-Å 3D map.

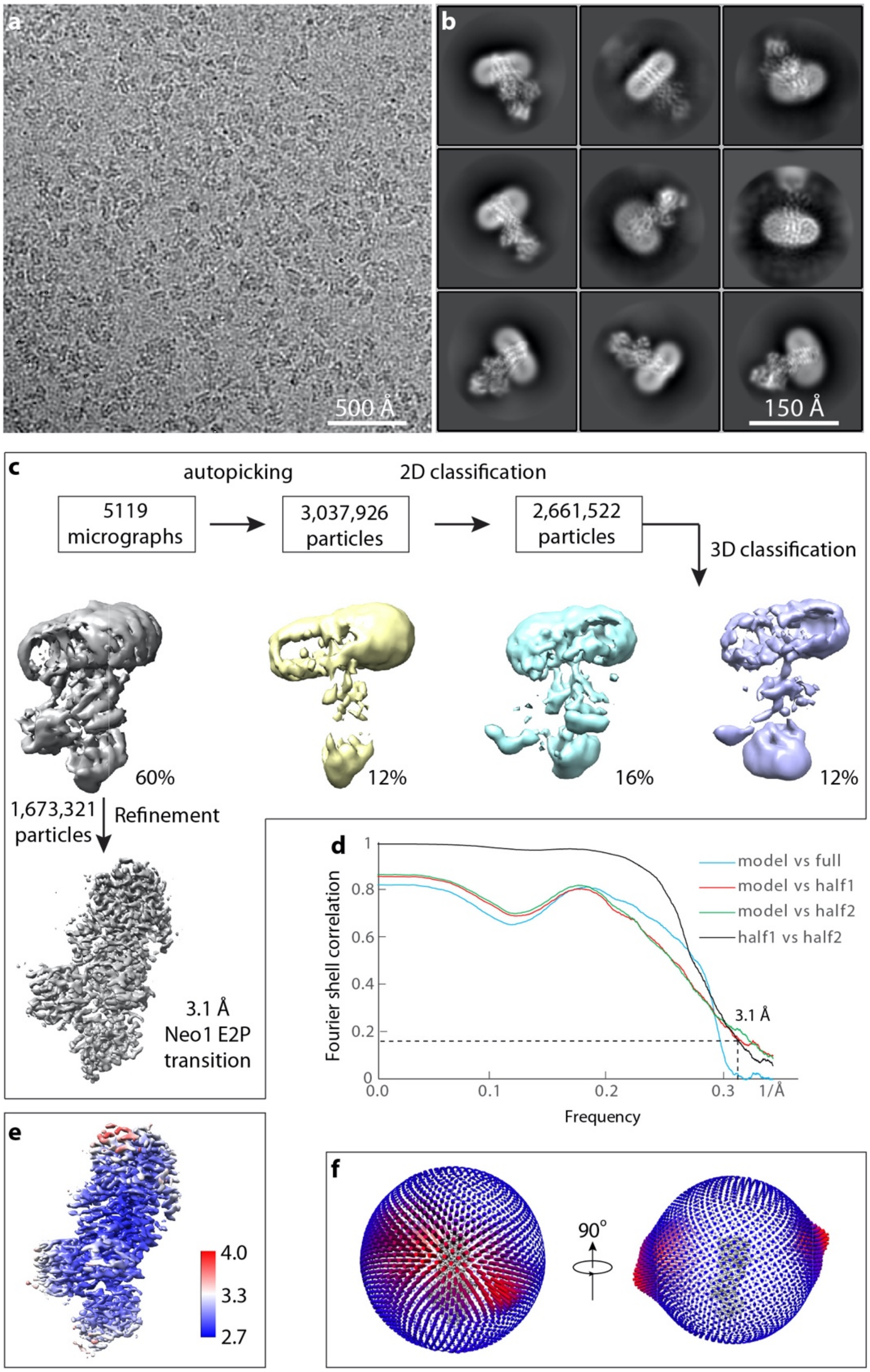

Supplemental Figure 3. **Cryo-EM data processing and validation of the Neo1 in the E2P-transition state. a**) A representative electron micrograph. **b**) Selected reference-free 2D class averages. **c**) Cryo-EM data processing procedure of Neo1 in the E2P transition state. **d**) Gold-standard Fourier shell correlation of two independent half 3D maps. **e**) Color-coded local resolution map. **f**) Euler angle distribution of particles used in final 3D reconstruction.

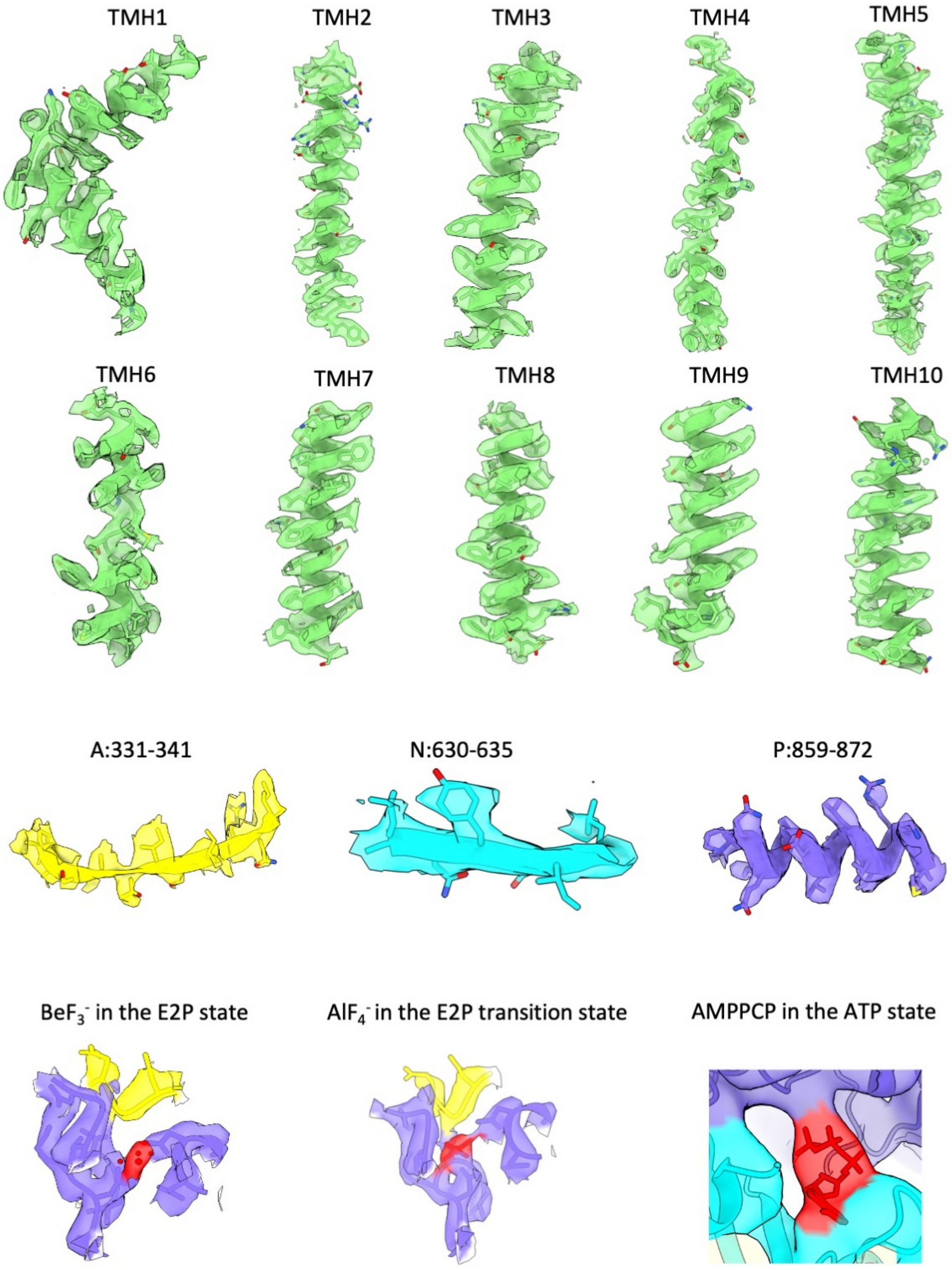

Supplemental Figure 4. Fit of the density map with the atomic model in several selected regions of the 3D map of Neo1 in the E2P state. Densities were surface rendered at a threshold of 5.6σ for TMH1-10 and A domain, 8.0σ for N and P domains, 7.8σ for BeF_3^-, 9.7σ for $AlF4^-$, and 5.5σ for AMPPCP.

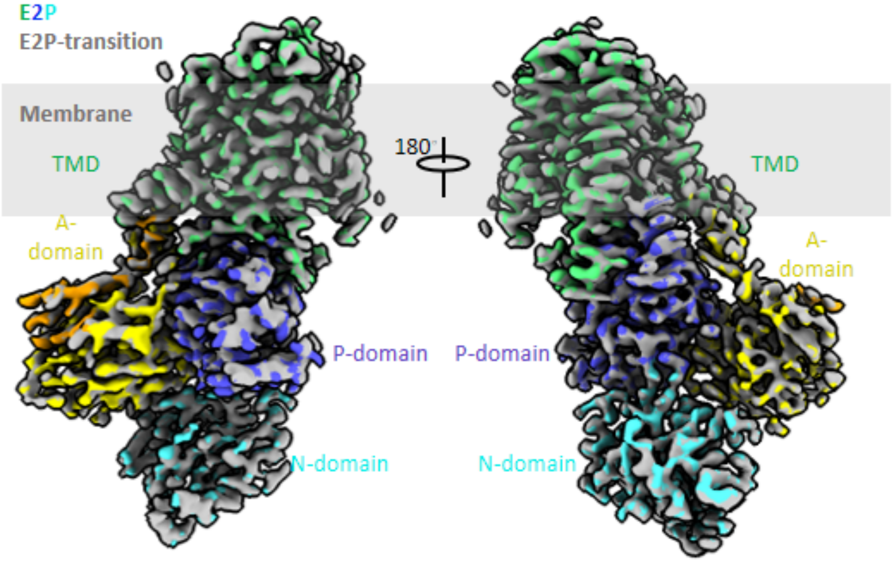

Supplemental Figure 5. Structural alignments between the Neo1 in the E2P-transition and the E2P states show a high similarity.

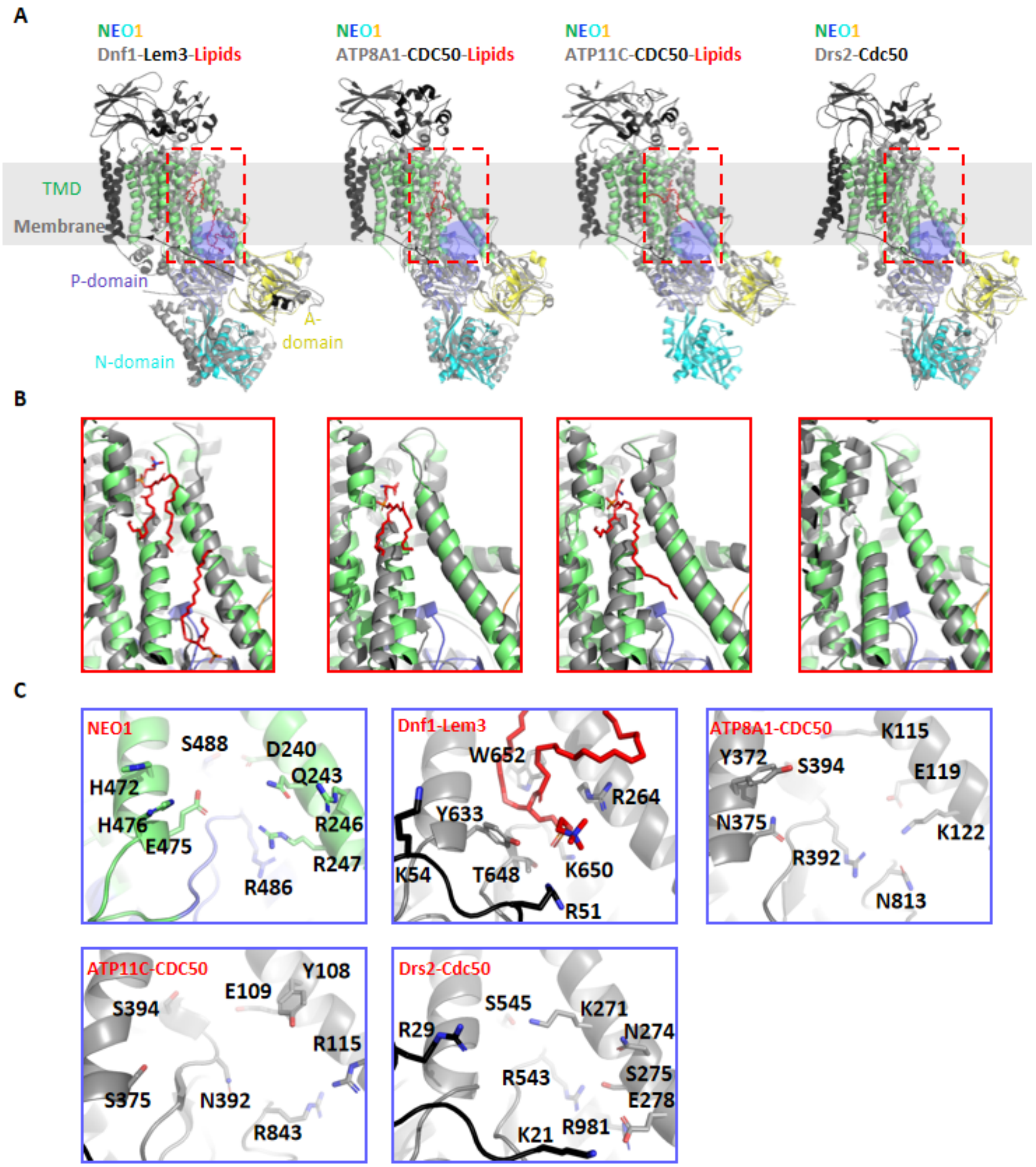

Supplemental Figure 6. Structural comparisons between the Neo1 in the E2P state and Dnf1–Lem3 (PDB ID 6K7M), ATP8A1-CDC50 (PDB ID 7KYC), ATP11C-CDC50 (PDB ID 7BSV), and Drs2-Cdc50 (PDB ID 6PSY). **a**) Neo1 structure superimposes well with Dnf1, ATP8A1, ATP11C, and Drs2. *Thermophilus* fugus Dnf1 is not shown here because it is nearly identical to the *S. cerevisiae* Dnf1. The red rectangles mark the substrate transport paths that are enlarged in pane (**b**). The bound lipids are shown as red sticks. **c**) Comparison of the substrate exit sites of five lipid flippases with known structures, emphasizing their conserved lipid-binding residues that are from both α- and β-subunits in most dimeric ATPases but only from the α-subunit in the single-subunit Neo1 ATPase. The β-subunits are shown in black.

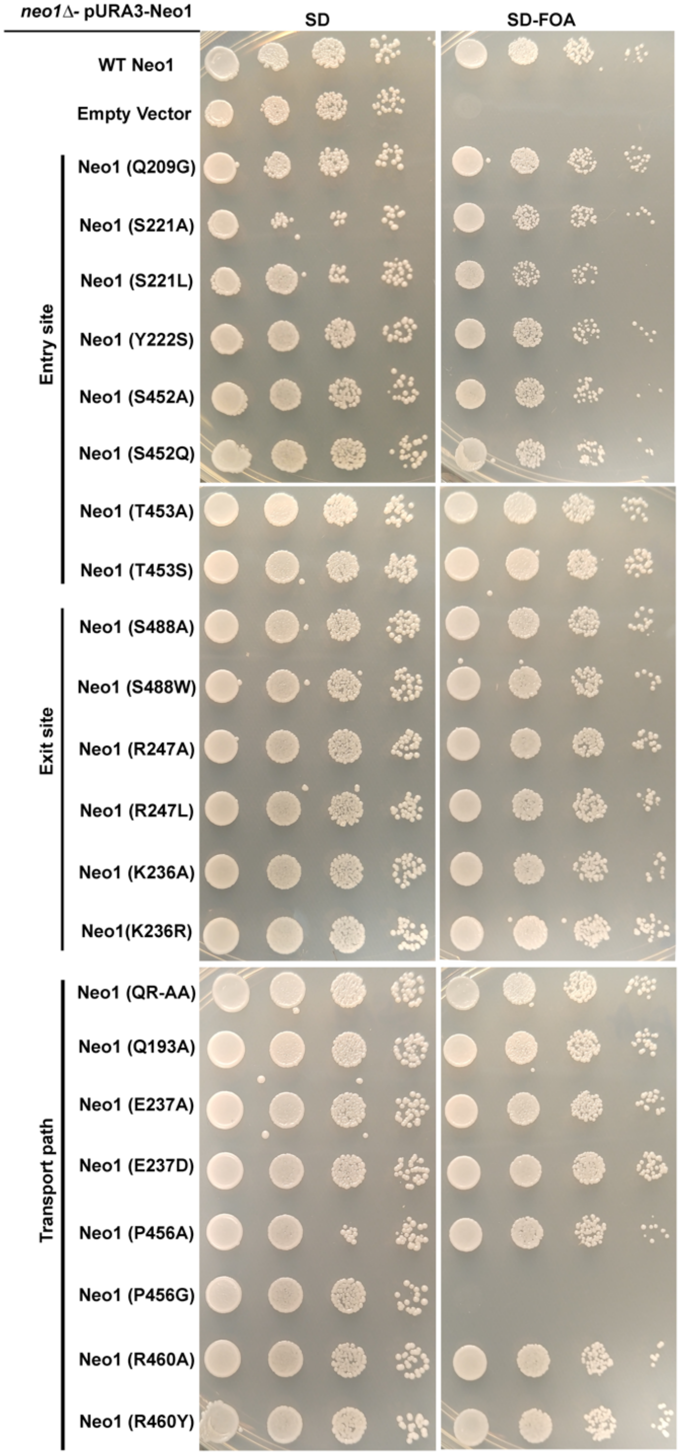

Supplemental Figure 7. **All of the substrate pathway Neo1 mutants except Neo1 P456G support viability of *neo1*Δ.** We transformed a *neo1*Δ *pURA3-NEO1* strain with HIS3-marked plasmids harboring the indicated Neo1 variant. Cells were spotted on SD to select both plasmids and on SD-5-FOA plates to kill cells that are unable to lose the *pURA3-NEO1* plasmid. *NEO1* is an essential gene, and all variants were able to complement the growth defect of *neo1*Δ except Neo1 P456G.

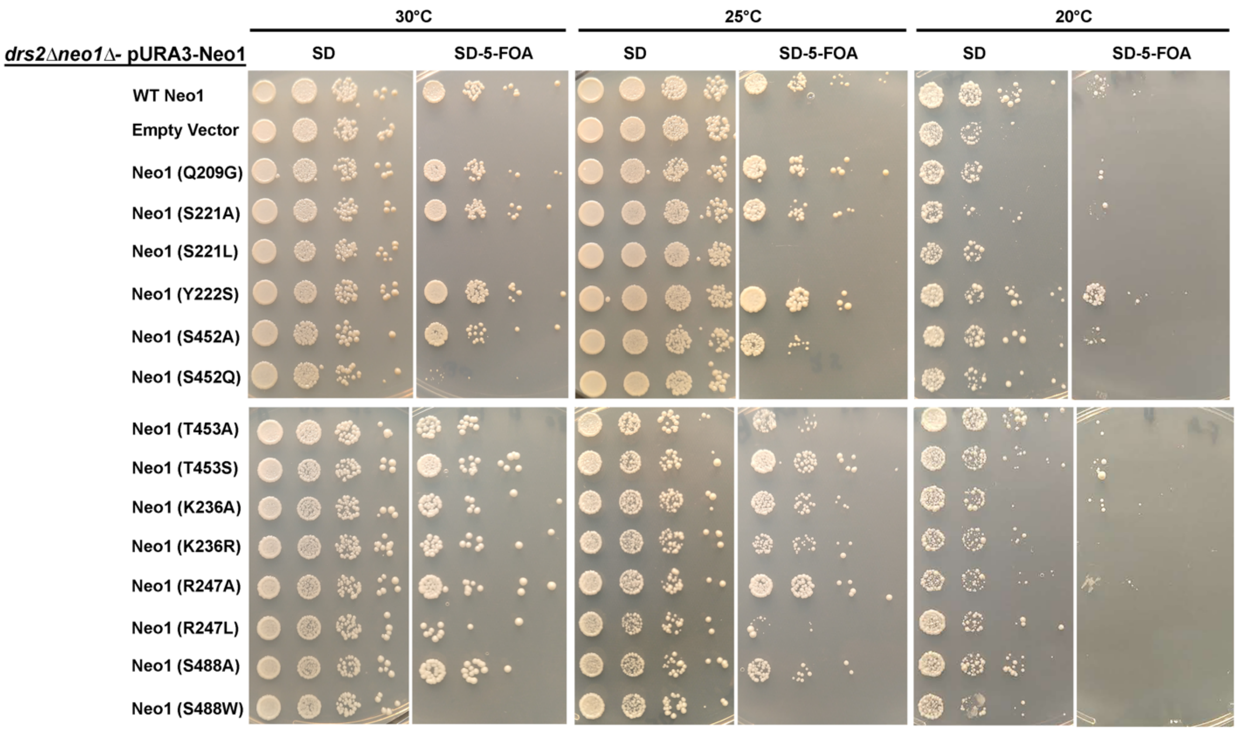

Supplemental Figure 8. Many of the Neo1 substrate pathway variants display synthetic growth defects with *drs2*∆*.* *Growth phenotypes for Neo1 mutants expressed in neo1∆drs2∆ strains at 30* °C*,* 25 °C *and 20* °C. Strains harboring *drs2*∆ are cold-sensitive for growth and fail to grow at 20 °C. As previously shown [57], Neo1 (Y222S) weakly suppressed the *drs2*∆ *cold-sensitive growth phenotype at 20* °C, but none of the other variants suppressed *drs2*∆*.*

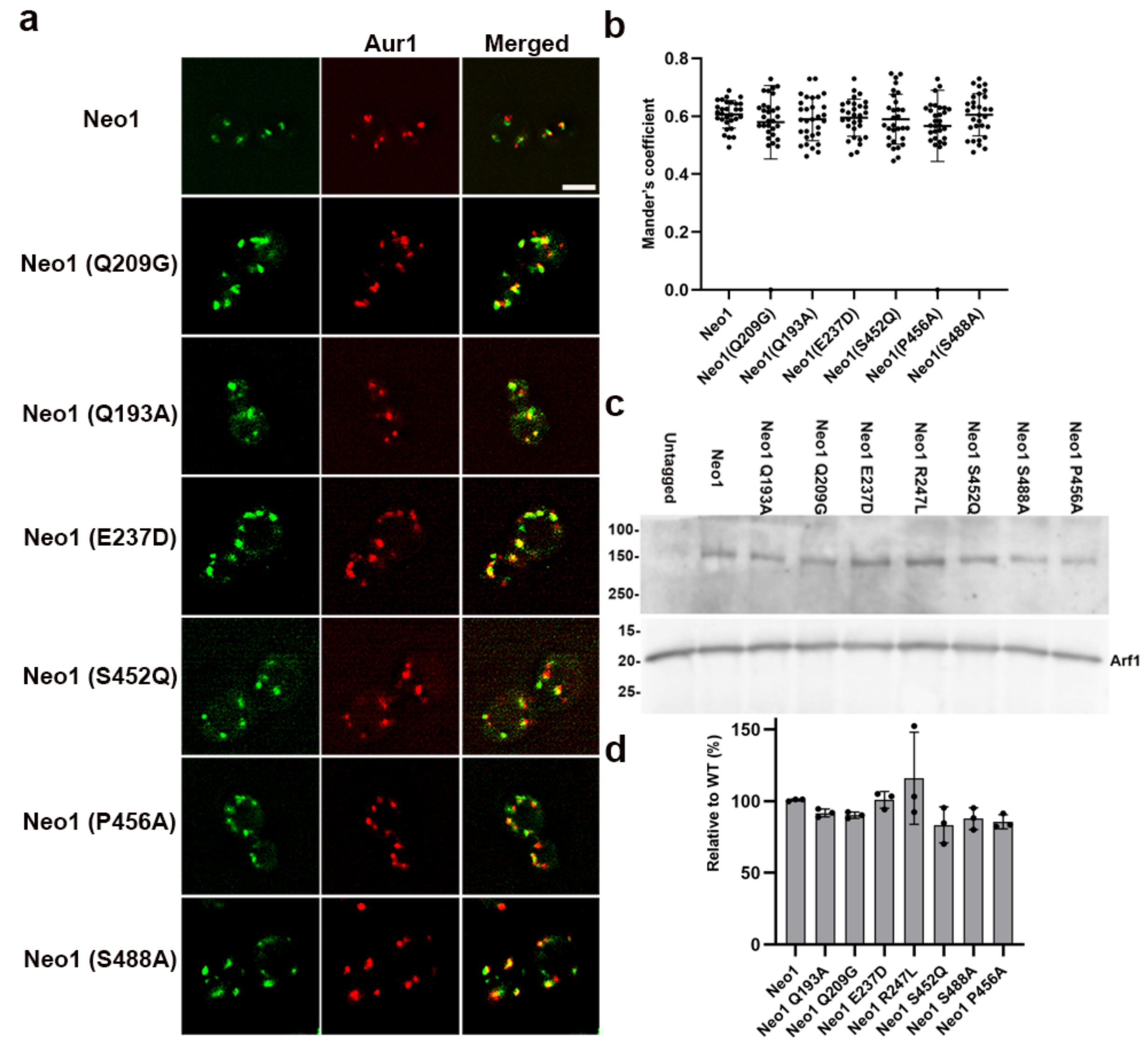

Supplemental Figure 9. Localization and expression of GFP-tagged Neo1 variants to the Golgi. **a**) GFP tagged Neo1 mutants were expressed in a strain expressing the medial Golgi marker mKate-Aur1 and imaged by fluorescence microscopy. Scale bar = 2 µm **b**) Manders' overlap coefficients were measured for GFP-Neo1 variants with the medial Golgi marker. For each variant, 30 cells were used to calculate the Manders' overlap coefficient. Data represent means ± SD (n=30). **c**) Expression and **d**) quantification of GFP tagged Neo1 variants by western blotting using anti-GFP. The wild-type strain (BY4741) expressing untagged Neo1 serves as a background control. For quantitation, the GFP-Neo1 band was quantified using ImageJ and normalized to the loading control Arf1 for each lane. Mutant Neo1 variants were plotted as the % of the WT GFP-Neo1 band intensity (n = 3). None of the Neo1 mutant variants were significantly different in localization or expression from the WT Neo1 control based on one-way ANOVA tests. The data indicates means ± SD.

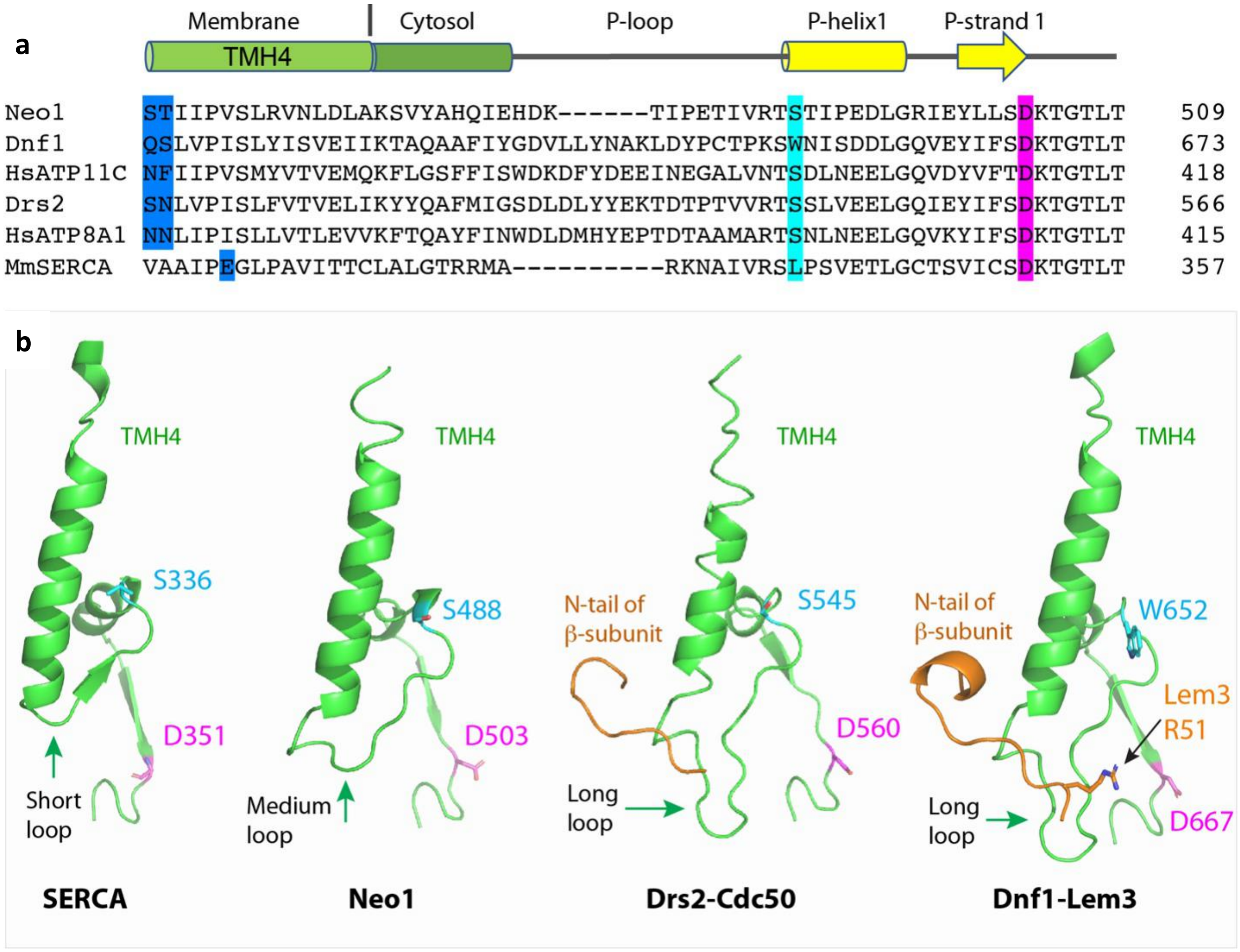

Supplemental figure 10. Extension of the TMH4 cytosolic loop in P4 ATPases relative to SERCA (P2 ATPAse). a) Alignment of TMH4 starting at entry site residues (marine), extending through the TMH4 cytosolic loop and into the P domain for the indicated P-type ATPases. **b)** Structures of the segments shown in the sequence alignment. These images emphasize the lengthening of the cytosolic loop coming out of TMH4 from P2 ATPases to P4B ATPases and P4A ATPases. The N-terminal tail of the β-subunit (orange) associates with this loop and in the case of Dnf1 helps position Lem3-R51 for substrate interaction. The crucial Neo1-S488 and Dnf1-W652 exit-gate residues (cyan) are positioned at the beginning of the P domain first α-helix and seem to form a substrate backstop that is closely linked to the phosphorylated aspartate (magenta residue).
